# Structure of semiconducting versus fast-ion conducting glasses in the Ag–Ge–Se system

**DOI:** 10.1098/rsos.171401

**Published:** 2018-01-17

**Authors:** Anita Zeidler, Philip S. Salmon, Dean A. J. Whittaker, Andrea Piarristeguy, Annie Pradel, Henry E. Fischer, Chris J. Benmore, Ozgur Gulbiten

**Affiliations:** 1 Department of Physics, University of Bath, Bath BA2 7AY, UK; 2 Institut Charles Gerhardt, UMR 5253 CNRS, CC 1503, Université de Montpellier, Pl. E. Bataillon, 34095 Montpellier Cedex 5, France; 3 Institut Laue Langevin, 71 Avenue des Martyrs, 38042 Grenoble Cedex 9, France; 4 X-ray Science Division, Advanced Photon Source, Argonne National Laboratory, 9700 South Cass Avenue, IL 60439, USA; 5 Science and Technology Division, Corning Incorporated, Corning, NY 14831, USA

**Keywords:** glass structure, phase separation, super-ionic phase, percolation transition, electric force microscopy, neutron and X-ray diffraction

## Abstract

The transition from a semiconductor to a fast-ion conductor with increasing silver content along the Ag_*x*_(Ge_0.25_Se_0.75_)_(100−*x*)_ tie line (0≤*x*≤25) was investigated on multiple length scales by employing a combination of electric force microscopy, X-ray diffraction, and neutron diffraction. The microscopy results show separation into silver-rich and silver-poor phases, where the Ag-rich phase percolates at the onset of fast-ion conductivity. The method of neutron diffraction with Ag isotope substitution was applied to the *x*=5 and *x*=25 compositions, and the results indicate an evolution in structure of the Ag-rich phase with change of composition. The Ag–Se nearest-neighbours are distributed about a distance of 2.64(1) Å, and the Ag–Se coordination number increases from 2.6(3) at *x*=5 to 3.3(2) at *x*=25. For *x*=25, the measured Ag–Ag partial pair-distribution function gives 1.9(2) Ag–Ag nearest-neighbours at a distance of 3.02(2) Å. The results show breakage of Se–Se homopolar bonds as silver is added to the Ge_0.25_Se_0.75_ base glass, and the limit of glass-formation at *x*≃28 coincides with an elimination of these bonds. A model is proposed for tracking the breakage of Se–Se homopolar bonds as silver is added to the base glass.

## 1. Introduction

The physico-chemical properties of chalcogenide glasses can be systematically manipulated by the addition of network modifiers [1]. An interesting case example is provided by glassy Ge–Se, where the addition of silver can lead to an abrupt transition in electrical behaviour from a semiconductor to a fast-ion conductor [2–7]. Microscopy studies suggest phase separation of the glass into domains that are either silver-rich or silver-poor, where the sharp increase in Ag-ion conductivity occurs at a composition for which the silver-rich phase percolates [5,8–12]. This ability of the glassy Ge–Se system to host silver has been exploited in programmable metallization cell technology for non-volatile computer memory, in which the application of a voltage between two electrodes fabricated on a solid electrolyte results in either the growth or dissolution of a metal filament between those electrodes [13–17]. Photo-induced migration of Ag also occurs, which gives these materials potential use as the sensing component in electronic dosimetry [18,19]. Numerous experimental and modelling studies have been performed to investigate the atomic scale structure of glassy Ag–Ge–Se materials [17,20–37]. However, a clear picture of the structure has yet to emerge, as befits the structural complexity.

**Figure 1.**
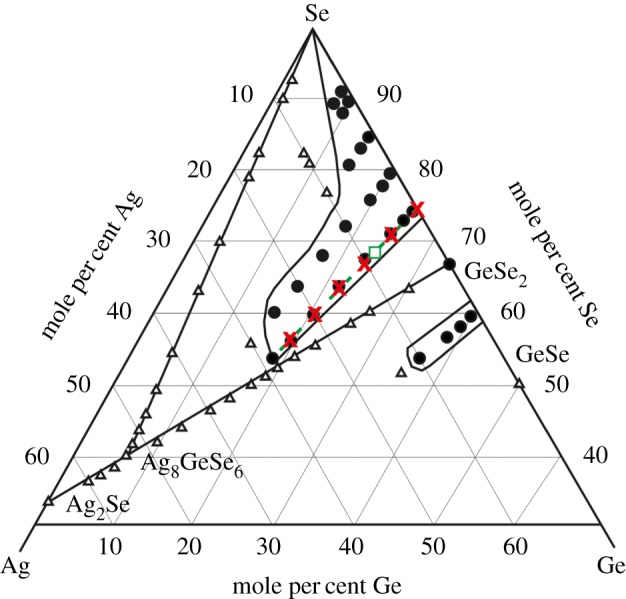
Glass formation in the ternary Ag–Ge–Se system, as adapted from Borisova *et al.* [38]. Glass forming compositions are identified by filled circles and compositions showing partial crystallinity are identified by open triangles. The broken (green) curve shows the Ag_*x*_(Ge_0.25_Se_0.75_)_100−*x*_ tie line, and the (green) square on this tie line marks the composition at which the glass becomes a fast-ion conductor with increasing Ag content [9]. The (red) crosses identify the glass compositions studied in this work.

The objective of this work is to explore the structure of glasses along the Ag_*x*_(Ge_0.25_Se_0.75_)_(100−*x*)_ tie line (0≤*x*≤25) [38] (figure 1), where the addition of silver leads to an abrupt semiconductor to fast-ion conductor transition at *x*≃8, and the total electrical conductivity jumps in value by 7–8 orders of magnitude to ≈10^−5^ *Ω*^−1^ cm^−1^ [2–6]. The glass structures are probed by using a combination of electric force microscopy (EFM), X-ray diffraction and neutron diffraction. The structures of the *x*=5 and *x*=25 compositions are also probed by applying the method of neutron diffraction with isotope substitution (NDIS). Here, Ag isotopes were employed, which enables the pair-correlation functions that describe the glass structure to be separated into two difference functions that describe either (i) the Ag–Ag and Ag–μ correlations or (ii) essentially the μ–μ^′^ correlations alone, where μ (or μ^′^) denotes a matrix (Ge or Se) atom. For the *x*=25 glass, the concentration of silver is sufficiently large to enable an identification of the relative distribution of Ag ions. As will be seen, the EFM experiments give information on the surface morphology of the glass and, assuming an absence of surface reconstruction, the results indicate phase separation of the bulk material. The diffraction results will therefore reveal a weighted average of the structures of the individual phases.

## 2. Theory

In a neutron or X-ray diffraction experiment on glass, the information on the material’s structure can be expressed by the total structure factor [39]

2.1 $$F(q)=\sum_{\alpha }\sum_{\beta }c_{\alpha }c_{\beta }b_{\alpha }(q)b_{\beta }^{*}(q)[S_{\alpha \beta }(q)-1],$$

where *c*_*α*_ and *c*_*β*_ are the atomic fractions of chemical species *α* and *β*, respectively, *b*_*α*_(*q*) and $b_{\alpha }^{*}(q)$ are the scattering length (or atomic form factor) and its complex conjugate for chemical species *α*, respectively, *q* is the magnitude of the scattering vector and *S*_*αβ*_(*q*) is a Faber–Ziman [40] partial structure factor. The latter is related to the partial pair-distribution function *g*_*αβ*_(*r*) via the Fourier transform relation

2.2 $$g_{\alpha \beta }(r)-1=\frac{1}{2\pi^{2}n_{0}r}\int_{0}^{\infty }dq[S_{\alpha \beta }(q)-1]q\sin(qr),$$

where *r* is a distance in real space, and *n*_0_ is the atomic number density. The mean coordination number of atoms of type *β*, contained within a spherical shell defined by radii *r*_*i*_ and *r*_*j*_ centred on an atom of type *α*, is given by

2.3 $${\bar{n}}_{\alpha }^{\beta }=4\pi n_{0}c_{\beta }\int_{r_{i}}^{r_{j}}dr\;g_{\alpha \beta }(r)r^{2}.$$

The scattering lengths are independent of *q* for the case of neutron diffraction, but not for the case of X-ray diffraction. To compensate for this *q* dependence, the total structure factor can be rewritten as

2.4 $$S(q)-1=\frac{F(q)}{|\langle b(q)\rangle {|}^{2}},$$

where the mean scattering length $\left\langle b(q)\rangle =\sum_{\alpha }c_{\alpha }b_{\alpha }(q\right)$.

The corresponding real-space information is given by the total pair-distribution function

2.5 $$g_{T}(r)-1=\frac{1}{2\pi^{2}n_{0}r}\int_{0}^{\infty }dq[S(q)-1]M(q)q\sin(qr),$$

where the modification function *M*(*q*) [*M*(*q*)=1 for *q*≤*q*_max_, *M*(*q*)=0 for *q*>*q*_max_] has been introduced to account for the fact that a diffractometer can access only a finite *q*-range. As q=(4π/λ)sin⁡θ, where λ is the incident neutron/photon wavelength and 2*θ* is the scattering angle [39], the cut-off maximum *q*_max_ is set by the wavelength and the maximum observable scattering angle. Provided *S*(*q*) no longer shows structure at *q*_max_, the effect of this finite cut-off can be neglected. Otherwise, each of the peaks in *g*_*αβ*_(*r*) that contribute towards *g*_T_(*r*) will be convolution broadened by the Fourier transform *M*(*r*) of the modification function *M*(*q*) [41]. At *r*-values smaller than the distance of closest approach between two atoms *g*_*αβ*_(*r*)=0, so the limiting value $g_{T}(r\to 0)=0$.

### 2.1. Neutron diffraction with isotope substitution

Consider three samples of glassy Ag–Ge–Se that are identical in every respect, apart from the isotopic enrichment of silver. Let the measured neutron total structure factors for samples containing ^Nat^Ag, ^107^Ag and ^109^Ag be denoted by ^Nat^*F*(*q*), ^107^*F*(*q*) and ^109^*F*(*q*), respectively, where Nat refers to the natural isotopic abundance of silver. In matrix notation it follows that

2.6 $$\left[\begin{array}{c} {}^{107}F(q)\\ {\;}^{\mathrm{Nat}}F(q)\\ {}^{109}F(q) \end{array}\right]=\left[\begin{array}{ccc} c_{\mathrm{Ag}}^{2}b_{{}^{107}\mathrm{Ag}}^{2} & 2c_{\mathrm{Ag}}b_{{}^{107}\mathrm{Ag}} & 1\\ c_{\mathrm{Ag}}^{2}b_{{\;}^{\mathrm{Nat}}\mathrm{Ag}}^{2} & 2c_{\mathrm{Ag}}b_{{\;}^{\mathrm{Nat}}\mathrm{Ag}} & 1\\ c_{\mathrm{Ag}}^{2}b_{{}^{109}\mathrm{Ag}}^{2} & 2c_{\mathrm{Ag}}b_{{}^{109}\mathrm{Ag}} & 1 \end{array}\right]\cdot \left[\begin{array}{c} S_{\mathrm{AgAg}}(q)-1\\ \Delta S_{\mathrm{Ag}\mu }(q)\\ \Delta S_{\mu \mu^{\prime }}(q) \end{array}\right],$$

where the difference function

2.7 $$\Delta S_{\mathrm{Ag}\mu }(q)=c_{\mathrm{Ge}}b_{\mathrm{Ge}}[S_{\mathrm{AgGe}}(q)-1]+c_{\mathrm{Se}}b_{\mathrm{Se}}[S_{\mathrm{AgSe}}(q)-1]$$

contains only Ag–μ correlations and has dimensions of length, and the difference function

2.8 $$\Delta S_{\mu \mu^{\prime }}(q)=\sum_{\alpha \neq \mathrm{Ag}}\sum_{\beta \neq \mathrm{Ag}}c_{\alpha }c_{\beta }b_{\alpha }b_{\beta }[S_{\alpha \beta }(q)-1]$$

contains only μ–μ^′^ correlations and has dimensions of area. If the Ag content of the glass is sufficiently high, equation (2.6) can be solved to deliver the silver–silver partial structure factor *S*_AgAg_(*q*) along with the difference functions Δ*S*_Agμ_(*q*) and Δ*S*_*μμ*′_(*q*).

The complexity of pair-correlations associated with a total structure factor can also be reduced by taking a difference between two total structure factors. For example, the μ–μ^′^ partial structure factors can be removed by taking a first difference function such as

2.9 $$\begin{array}{rl} \Delta F_{\mathrm{Ag}}(q) & ={}^{107}F(q)-{}^{109}F(q)\\ & =c_{\mathrm{Ag}}^{2}(b_{{}^{107}\mathrm{Ag}}^{2}-b_{{}^{109}\mathrm{Ag}}^{2})[S_{\mathrm{AgAg}}(q)-1]\\ & \;+2c_{\mathrm{Ag}}(b_{{}^{107}\mathrm{Ag}}-b_{{}^{109}\mathrm{Ag}})\Delta S_{\mathrm{Ag}\mu }(q). \end{array}$$

Likewise, the Ag–μ correlations can be removed by taking a weighted difference function such as

2.10 $$\begin{array}{rl} \Delta F(q) & =[b_{{}^{107}\mathrm{Ag}}{}^{109}F(q)-b_{{}^{109}\mathrm{Ag}}{}^{107}F(q)]/(b_{{}^{107}\mathrm{Ag}}-b_{{}^{109}\mathrm{Ag}})\\ & =\Delta S_{\mu \mu^{\prime }}(q)-c_{\mathrm{Ag}}^{2}b_{{}^{107}\mathrm{Ag}}b_{{}^{109}\mathrm{Ag}}[S_{\mathrm{AgAg}}(q)-1]. \end{array}$$

The *r*-space functions corresponding to *F*(*q*), Δ*F*_Ag_(*q*), Δ*S*_Agμ_(*q*), Δ*F*(*q*) and Δ*S*_*μμ*′_(*q*) are obtained by Fourier transformation and are denoted by *G*(*r*), Δ*G*_Ag_(*r*), Δ*G*_Agμ_(*r*), Δ*G*(*r*) and Δ*G*_*μμ*′_(*r*), respectively. The equation for a given *r*-space function is obtained from that of the corresponding *q*-space function by replacing each partial structure factor *S*_*αβ*_(*q*) by the matching partial pair-distribution function *g*_*αβ*_(*r*). The theoretical low-*r* limits then follow from setting $g_{\alpha \beta }(r\to 0)=0$. For example, the total pair-distribution function $G(r)={\left\langle b\right\rangle }^{2}[g_{T}(r)-1]$ [39], so $G(r\to 0)=-{\left\langle b\right\rangle }^{2}$.

## 3. Material and methods

### 3.1. Sample preparation

To remove oxygen impurities, powdered silver metal (greater than or equal to 99.9%, Sigma Aldrich) was processed in a stream of hydrogen gas within a reduction furnace at a temperature of 400^°^C for 14–19 h. The metal was then transferred to a high-purity argon-filled glove box under inert gas conditions. The glassy samples of ^Nat^Ag_*x*_(Ge_0.25_Se_0.75_)_(100−*x*)_ (of mass ≈3 g) were prepared in this glove box by loading Ag, Ge (99.999%, Sigma Aldrich), and Se (greater than or equal to 99.999%, Sigma Aldrich), in the desired mass ratio, into silica ampoules of 5 mm inner diameter and 1 mm wall thickness. The ampoules had been cleaned by etching with a 48 wt% aqueous solution of hydrofluoric acid, rinsed with distilled water then acetone, dried and then baked-out under a vacuum of ≈10^−5^ Torr for 2–4 h at 800^°^C. The loaded ampoules were evacuated to ≈10^−5^ Torr for ≈14 h, sealed, and then placed into a rocking furnace. The temperature was increased at 1^°^C min^−1^ to 962^°^C (the melting point of Ag), dwelling for 4 h each at 221^°^C (the melting point of Se), 685^°^C (the boiling point of Se) and 938^°^C (the melting point of Ge). The upper temperature was maintained for 18 h, after which the rocking motion was stopped, and the furnace was set vertically to allow liquid to collect at the bottom of the ampoule. After a further 6 h, the temperature was decreased at 1^°^C min^−1^ to 800^°^C, which was maintained for 5 h, and the samples were then quenched by dropping the ampoules into an ice–water mixture. The same procedure was also used to prepare the *x*=25 samples containing ^107^Ag (99.50% enrichment, Isoflex) and ^109^Ag (99.40% enrichment, Isoflex), and included the removal of oxygen impurities from the silver metal.

After the neutron diffraction patterns for the *x*=25 samples were measured, Ge and Se were added to make the samples used in the neutron diffraction experiments described in [36]. Subsequently, more Ge and Se were added to make the *x*=5 samples described in this work. Each sample was prepared using the heating and cooling procedure described above. The coherent neutron scattering lengths of the elements, taking into account the enrichment of the silver isotopes, are listed in table 1.

**Table 1.** The coherent neutron scattering lengths of the elements [42], taking into account the enrichment of the silver isotopes.

*b*_Ge_/fm	*b*_Se_/fm	*b*_^Nat^Ag_/fm	$b_{{}^{107}\mathrm{Ag}}$/fm	$b_{{}^{109}\mathrm{Ag}}$/fm
8.185(20)	7.970(9)	5.922(7)	7.538(11)	4.185(11)

### 3.2. Mass density

**Figure 2.**
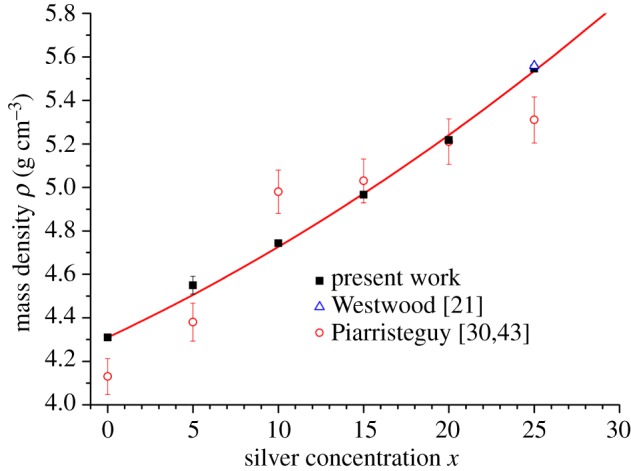
The composition dependence of the mass density along the Ag_*x*_(Ge_0.25_Se_0.75_)_(100−*x*)_ tie line as measured in this work (filled squares), and in the previous work of Piarristeguy *et al.* [30,43] (open (red) circles) and Westwood *et al.* [21] for *x*=25 (open (blue) triangle). A least-squares fit to the results of this work gives *ρ*(*g* *cm*^−3^)=4.309(3)+0.036(2)*x*+4.9(8)*x*^2^ (solid (red) curve).

Densities were measured using a Quantachrome MICRO-ULTRAPYC 1200*e* pycnometer operated with He gas at a temperature of 21^°^C. For each sample, ≈150 measurements were taken, and the statistical uncertainty was obtained by finding the standard deviation about the mean. The results are shown in figure 2, where they are compared to those obtained from previous work [21,30,43].

### 3.3. Electric force microscopy

**Figure 3.**
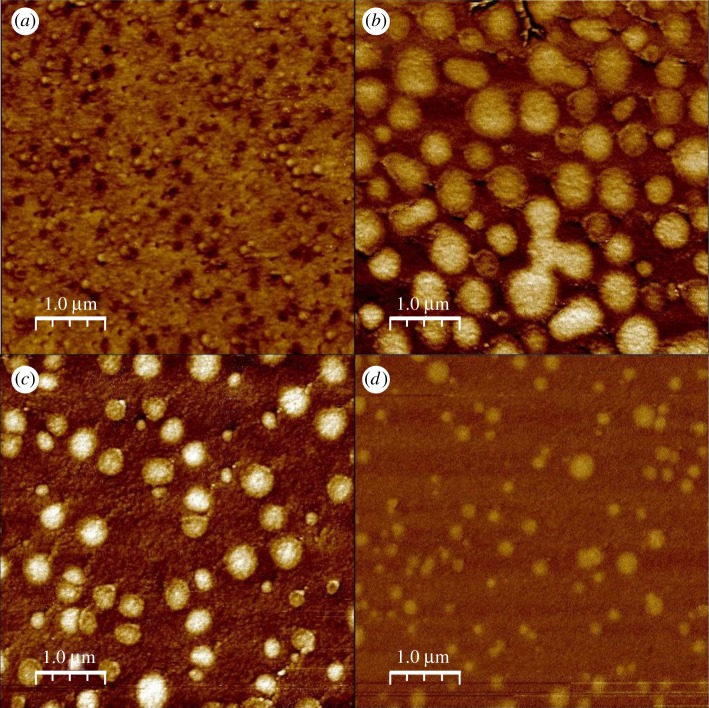
EFM images for glasses along the Ag_*x*_(Ge_0.25_Se_0.75_)_(100−*x*)_ tie line, where (*a*) *x*=5, (*b*) *x*=15, (*c*) *x*=20 or (*d*) *x*=25. The images in (*a*–*c*) were taken with an applied voltage *V* =−4 V, and the image in (*d*) was taken with *V* =−2 V. The dark and light patches show regions of high- and low-Ag content, respectively.

It is difficult to accurately measure the microstructure of silver containing glasses using standard techniques, such as scanning electron microscopy coupled with energy dispersive X-ray spectroscopy or electron probe microanalysis, because of the high mobility of silver, and the sensitivity of this mobility to the flux of photons or electrons used as the probe [12]. It is, therefore, desirable to use a methodology that will not induce local structural modifications. The EFM method offers this advantage, and allows the electrical heterogeneity at the surface of glass to be measured by probing changes to the electric permittivity [8,12]. An electric field is generated between the tip of a cantilever and the glass surface by applying a voltage *V* , and the oscillation frequency of the cantilever is affected by the tip–sample interaction, which depends on the electrical state of the sample surface [12]. The experiments were performed under ambient conditions, using a Veeco Dimension 3100 scanning probe microscope, on the surfaces of freshly fractured glass to avoid contamination by oxidation. The microscope was operated using a conventional frequency modulation technique at the first cantilever frequency (60 kHz) using a commercial coated (PtIr5) cantilever tip in lift-mode at a distance 30 nm above the sample surface. The applied voltage *V* was chosen to optimize the image contrast. Further details are given in [12]. Several of the images are presented in figure 3, and show phase separation. For the semiconducting regime at *x*=5, Ag-rich regions of size d≲0.25 μm are isolated by Ag-poor regions. The converse is true for the fast-ion conducting regime at *x*=15, where silver-poor regions of size 0.25≲d (μm) ≲1 are isolated by Ag-rich regions. With further increase of Ag content, the Ag-poor regions diminish as the Ag-rich regions grow in size.

### 3.4. Differential scanning calorimetry

**Figure 4.**
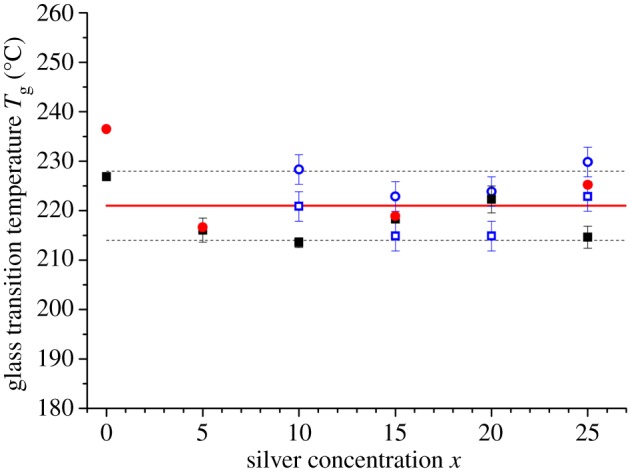
The composition dependence of the glass transition temperature *T*_g_ along the Ag_*x*_(Ge_0.25_Se_0.75_)_(100−*x*)_ tie line as measured using differential scanning calorimetry with scan rates of 3^°^C min^−1^ (filled squares) or 50^°^C min^−1^ (filled (red) circles). The solid (red) curve shows the overall mean $\left\langle T_{g}\right\rangle =221^{\circ }$C, and the broken curves indicate the standard deviation of ±7^°^C. Also shown are the *T*_g_ values reported in [5,44] for scan rates of 10^°^C min^−1^ (open (blue) squares) or 80^°^C min^−1^ (open (blue) circles).

The glass transition temperature *T*_g_ was measured using a TA Instruments Q100 differential scanning calorimeter, operated in temperature modulation mode with a scan rate of 3^°^C min^−1^ and modulation of ±1^°^C per 100 s. The samples, of mass approximately 20 mg, were loaded into crimped Al pans, and oxygen-free nitrogen was used as the purge gas with a flow rate of 50 ml min^−1^. In addition, *T*_g_ was measured for selected compositions using an inter-cooler equipped Mettler Toledo DSC2 calorimeter with a scan rate of 50^°^C min^−1^, after each sample had been temperature cycled by heating at a rate of 50^°^C min^−1^ to the supercooled liquid above *T*_g_ and quenching at the same rate. The composition dependence of *T*_g_, as taken from the onset of the glass transition in the total heat flow, is shown in figure 4. The results show little deviation with composition from a mean value $\left\langle T_{g}\right\rangle =221{\left(7\right)}^{\circ }$C, so it was not possible to detect phase separation from the calorimetry experiments. This finding is consistent with other differential scanning calorimetry work on glasses along the Ag_*x*_(Ge_0.25_Se_0.75_)_(100−*x*)_ tie line [2,5,44]. The *T*_g_ values from this work are consistent with those reported in [5,44] (figure 4), but a wider spread of values is given in [2]. Wang *et al.* [45] report two *T*_g_ values from modulated differential scanning calorimetry measurements, but it was necessary to partially crystallize the material before a second *T*_g_ could be observed, i.e. only a single *T*_g_ was observed in the absence of crystallization.

### 3.5. Neutron diffraction

The neutron diffraction experiments were performed in two parts. In each, the D4c instrument at the Institut Laue-Langevin in Grenoble [46] was employed to measure the diffraction pattern for each sample in a vanadium container of inner diameter 4.8 mm and wall thickness 0.1 mm; the empty vanadium container; the empty instrument; a vanadium rod of diameter 6.078(2) mm for normalization purposes; and an absorbing ^10^B_4_C bar in order to correct for the effect of sample attenuation on the background count-rate at small scattering angles. Counting times were optimized using the procedure described in [47]. The incident neutron wavelength was λ=0.4978(1) Å, except for the NDIS experiments on the *x*=5 composition where λ=0.6950(1) Å. The diffractometer benefited from a higher neutron flux at this longer wavelength, which leads to a smaller *q*_max_ value.

The data analysis followed the procedure described elsewhere [48]. Self-consistency checks were performed to ensure that (i) each neutron total structure factor *S*_N_(*q*) obeys the sum-rule relation $\int_{0}^{\infty }dq\;q^{2}[S_{N}(q)-1]=-2\pi^{2}n_{0}$, which follows from equation (2.5) by neglecting the effect of *M*(*q*) and taking the limit as r→0; (ii) the low-*r* features in the corresponding neutron total pair-distribution function *g*_T,N_(*r*) oscillate about the theoretical limit $g_{T,N}(r\to 0)=0$; and (iii) the back Fourier transform of *g*_T,N_(*r*), after the unphysical low-*r* oscillations are set to the limiting value $g_{T,N}(r\to 0)=0$, is in good overall agreement with the measured *S*_N_(*q*) function [48].

### 3.6. X-ray diffraction

The high-energy X-ray diffraction experiments employed beamline 11-ID-C at the Advanced Photon Source, Argonne National Laboratory in Chicago. A Perkin-Elmer model XRD 1621 CN3 EHS amorphous-silicon flat-plate area-detector (pixel size of 200×200 μm) was mounted perpendicular to the incident beam at a distance of 380 mm from the sample. The incident photon energy was 115 keV, and the incident beam had a square profile of side-length 0.5 mm. The samples were loaded into Kapton^®^ tubes (from Cole-Palmer) of 1.27 mm inner diameter and 0.05 mm wall thickness within an argon filled glovebox, and the tubes were sealed with Araldite^®^. X-ray diffraction patterns were measured for each sample in its container, an empty container, and a powdered CeO_2_ sample for detector calibration purposes. To test for reproducibility, two different parts of each sample were studied by moving the sample on an *x*–*y* stage. To correct for a background signal produced by the detector electronics, a ‘dark’ pattern was collected after each measurement with the beam off. The two-dimensional diffraction data were integrated using FIT2D [49,50]. The atomic form factors used in the data analysis were taken from Waasmaier & Kirfel [51], and the Compton scattering corrections for Ag and for Ge and Se were taken from Cromer & Mann [52] and Cromer [53], respectively.

## 4. Results

### 4.1. Neutron and X-ray total structure factors

**Figure 5.**
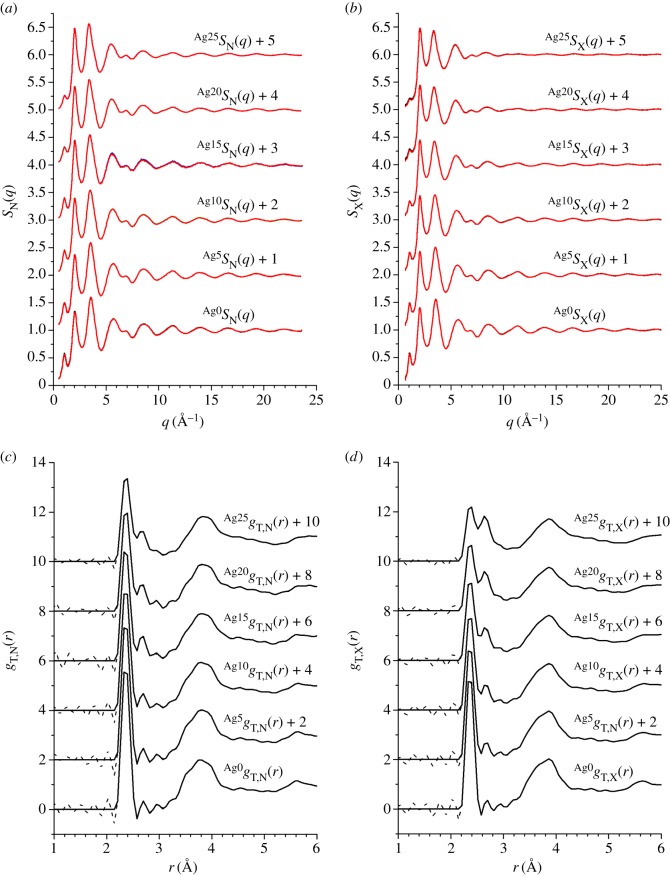
The measured (*a*) neutron *S*_N_(*q*) and (*b*) X-ray *S*_X_(*q*) total structure factors at a temperature ≈25^°^C for glasses containing Ag of natural isotopic abundance along the Ag_*x*_(Ge_0.25_Se_0.75_)_(100−*x*)_ tie line. The compositions are indicated by the superscripts Agx, where *x*=0, 5, 10, 15, 20 or 25. The solid (black) curves with vertical error bars denote the measured functions, where the size of an error bar is smaller than the curve thickness at most *q* values. The light solid (red) curves are the back Fourier transforms of the real-space functions *g*_T,N_(*r*) and *g*_T,X_(*r*) shown in (*c*) and (*d*), respectively, after the unphysical low-*r* oscillations shown by the broken curves are set to the theoretical $g_{T,N}(r\to 0)$ or $g_{T,X}(r\to 0)$ limit.

**Figure 6.**
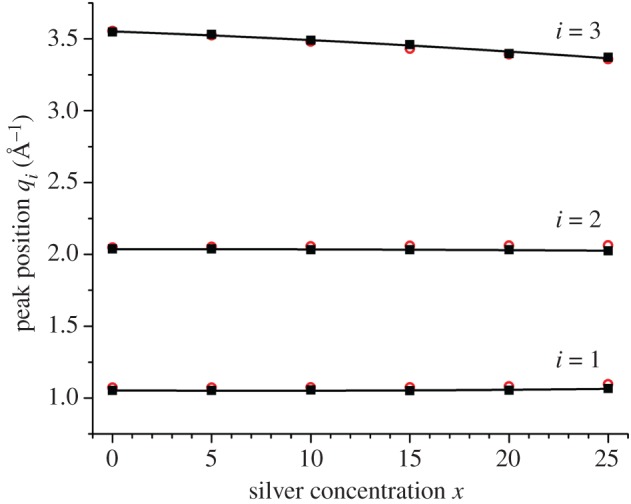
The composition dependence of the first three peak positions *q*_*i*_ (*i*=1, 2 or 3) in *S*_N_(*q*) (filled squares) or *S*_X_(*q*) (open (red) circles) for glassy samples of ^Nat^Ag_*x*_(Ge_0.25_Se_0.75_)_(100−*x*)_. The curves are drawn as guides for the eye.

The measured neutron *S*_N_(*q*) and X-ray *S*_X_(*q*) total structure factors are shown in figure 5*a*,*b*, respectively. For a given composition, the *S*_N_(*q*) and *S*_X_(*q*) functions are similar, and the first three peak positions *q*_*i*_ (*i*=1, 2 or 3) are the same within the experimental error (figure 6). The first and second peaks at *q*_1_ and *q*_2_ are often referred to as the first sharp diffraction peak (FSDP) and principal peak, respectively. The real-space periodicity 2*π*/*q*_*i*_ originating from each peak is associated with ordering on a length scale that is commensurate with the nearest-neighbour separations (*q*_3_), with the size of a local network-forming motif (*q*_2_), or with the arrangement of these motifs on an intermediate range (*q*_1_) [54]. For both the neutron and X-ray data sets, the heights of the FSDP at *q*_1_≃1.06 Å^−1^ and the third peak at *q*_3_≃3.55 Å^−1^ decrease with increasing silver content, whereas the height of the principal peak at *q*_2_≃2.04 Å^−1^ increases.

The neutron *g*_T,N_(*r*) and X-ray *g*_T,X_(*r*) total pair-distribution functions are shown in figure 5*c*,*d*, respectively. In both cases, the first peak at 2.37(1) Åis likely to originate from a combination of Ge–Se and Se–Se correlations, as found from the measured set of *g*_*αβ*_(*r*) functions for the Ge_0.25_Se_0.75_ base glass [55]. A second peak at ≃2.64 Åemerges with increasing silver content and, by comparison with the structures of the crystalline polymorphs of Ag_8_GeSe_6_ [56–58], it is attributed to nearest-neighbour Ag–Se correlations. The peak at ≃2.64 Å is more prominent in *g*_T,X_(*r*) when compared with *g*_T,N_(*r*), which originates from the large X-ray atomic form factor for Ag, i.e. the silver pair-distribution functions receive a larger weighting in *g*_T,X_(*r*) when compared with *g*_T,N_(*r*). For *x*=0, a shoulder on the low-*r* side of the peak at ≃3.8 Å, which is attributed to corner-sharing Ge–Ge correlations by comparison with the measured set of *g*_*αβ*_(*r*) functions for the base glass [55], becomes less pronounced with increasing silver content.

To obtain additional information on the local structure, it is necessary to take into account the effect of the finite *q*_max_ value of the diffractometer on the measured real-space functions. The first few peaks in $D_{T,N}(r)\equiv 4\pi n_{0}r[g_{T,N}(r)-1]=4\pi n_{0}rG_{N}(r)/|G_{N}(r\to 0)|$ over the range 2–3.2 Åwere therefore fitted to a sum of five Gaussian functions, each convoluted with the Fourier transform *M*(*r*) of the modification function *M*(*q*) [41]. A Gaussian function in *D*_T,N_(*r*) is symmetrically broadened by *M*(*r*). The neutron diffraction results were chosen for this analysis because the coherent neutron scattering lengths are *q*-independent, leading to a relatively simple real-space fitting procedure. For the crystalline polymorphs of Ag_8_GeSe_6_, Ge is bound to 4 Se atoms, Ag is bound to 3 or 4 Se atoms, the nearest-neighbour Ag–Ag distance is ≃3 Åand the shortest Ag–Ge distances are in the range 3.70–3.91 Å[56–58]. In the *β*^′^-Ag_8_GeSe_6_ phase, for example, Ag has 3 or 4 Se atoms at distances in the range 2.53–2.91 Å, the nearest-neighbour Ag–Ag distances are in the range 2.99–3.18 Å and the shortest Ag–Ge distance is 3.70 Å [58]. In the Ge_0.25_Se_0.75_ base glass, the measured set of *g*_*αβ*_(*r*) functions show both Ge–Se and Se–Se nearest-neighbours, with the bond distances and coordination numbers summarized in table 2 [55].

**Table 2.** The nearest-neighbour μ–μ^′^ distances and coordination numbers extracted from neutron diffraction experiments on glasses along the Ag_*x*_(Ge_0.25_Se_0.75_)_(100−*x*)_ tie line. Results are also given from NDIS experiments on the Ge_0.25_Se_0.75_ base glass [55], from previous neutron diffraction [24,29] and anomalous X-ray scattering [21] experiments on the silver-modified glass, from an analysis of anomalous X-ray scattering data using the reverse Monte Carlo method [34,35], and from a previous NDIS experiment on a related glass [36].

*x*	*r*_GeSe_ (Å)	*r*_SeSe_ (Å)	${\bar{n}}_{\mathrm{Ge}}^{\mathrm{Se}}$	${\bar{n}}_{\mathrm{Se}}^{\mathrm{Se}}$	function	reference
0	2.37(1)	2.36(1)	4	0.66(5)	*D*_T,N_(*r*)	this work
	2.37(2)	—	4.00(2)	—	*g*_GeSe_(*r*)	[55]
	—	2.35(2)	—	0.70(2)	*g*_SeSe_(*r*)	[55]
4.2	2.35	2.35	4.07	0.78	—	[35]
5	2.37(1)	2.35(1)	4	0.66(5)	*D*_T,N_(*r*)	this work
	2.37(1)	2.36(1)	4	0.66(5)	Δ*D*(*r*)	this work
7.7^a^	2.37(1)	2.36(1)	4	0.81(4)	Δ*D*(*r*)	[36]
10	2.37(1)	2.36(1)	4	0.59(5)	*D*_T,N_(*r*)	this work
15	2.37(1)	2.35(1)	4	0.50(5)	*D*_T,N_(*r*)	this work
	2.38	2.38	4	0.9	—	[29]
20	2.37(1)	2.36(1)	4	0.45(5)	*D*_T,N_(*r*)	this work
	2.35	2.45	3.98	0.65	—	[35]
25	2.37(1)	2.36(1)	4	0.19(5)	*D*_T,N_(*r*)	this work
	2.37(1)	2.36(1)	4	0.24(5)	Δ*D*(*r*)	this work
	2.37(1)	2.35(1)	4	0.05(5)	Δ*D*_*μμ*^′^_(*r*)	this work
	2.37(1)	—	4.01(5)	—	—	[24]
	2.38	2.38	4	0.7	—	[29]
	2.38	—	3.83	0.45	—	[21]
	2.35	2.55	4.01	0.65	—	[34]

^a^Corresponds to *x*=7.7 on the Ag_*x*_(Ge_0.23_Se_0.77_)_(100−*x*)_ tie line [36].

**Figure 7.**
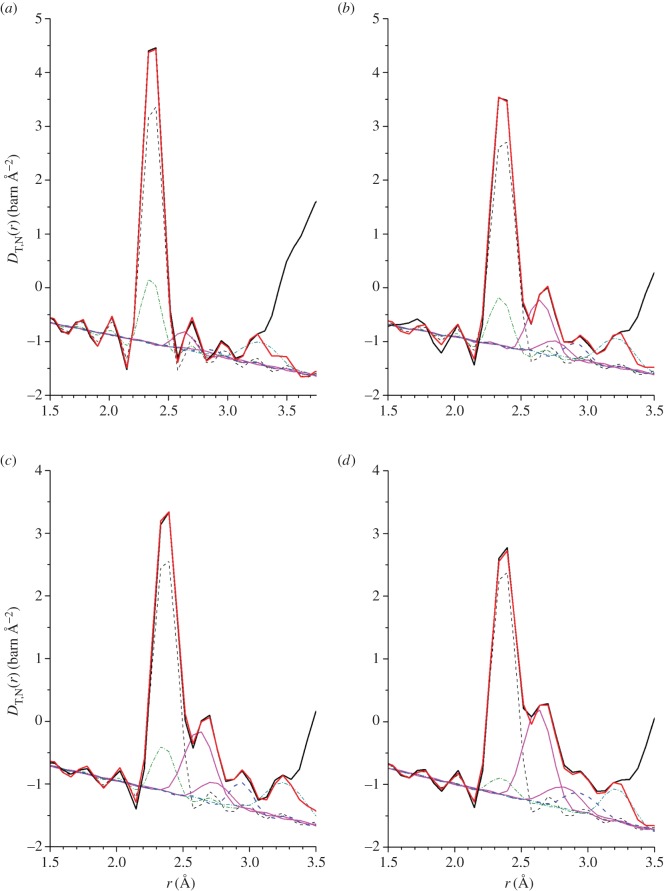
The fitted *D*_T,N_(*r*) functions for (*a*) *x*=5, (*b*) *x*=15, (*c*) *x*=20 and (*d*) *x*=25. The measured data sets are shown by the solid (black) curves, and the sums of fitted Gaussian functions are shown by the light solid (red) curves. The peak at 2.37 Å was fitted using two Gaussian functions, representing Ge–Se (broken (black) curve) and Se–Se (chained (green) curve) correlations, respectively. The peak at ≈2.67 Å was fitted using two Gaussian functions, representing Ag–Se correlations (solid (magenta) curves), and the third peak at ≈2.9 Å was fitted using a single Gaussian function, representing Ag–Ag correlations (broken (blue) curve). The Gaussian function at the largest *r*-value (chained (cyan) curve) was used as a constraint on fitting the peaks at lower-*r* values.

The first peak in *D*_T,N_(*r*) was therefore fitted using two Gaussian functions representing the nearest-neighbour Ge–Se and Se–Se correlations, with the Ge–Se coordination number constrained to give the base-glass value ${\bar{n}}_{\mathrm{Ge}}^{\mathrm{Se}}=4$. The presence of GeSe_4_ tetrahedral motifs is supported by Raman spectroscopy experiments on glasses along the Ag_*x*_(Ge_0.25_Se_0.75_)_(100−*x*)_ tie line [25], and by Raman spectroscopy and inelastic neutron scattering experiments on the *x*=25 glass [59]. Ag–Se nearest-neighbours will appear at larger *r*-values, so the third and fourth fitted Gaussian functions were attributed to Ag–Se correlations. The fifth fitted Gaussian was assigned to Ag–Ag nearest-neighbours, giving a minimum Ag–Ag distance ${\bar{r}}_{\mathrm{AgAg}}≃3$ Å. Several of the fitted *D*_T,N_(*r*) functions are shown in figure 7. The associated peak positions and coordination numbers are listed in table 2 for the μ–μ^′^ correlations, and in table 3 for the Ag–μ and Ag–Ag correlations. On the premise that ${\bar{n}}_{\mathrm{Ge}}^{\mathrm{Se}}=4$, Se–Se homopolar bonds are necessary to account for the area under the first peak in *D*_T,N_(*r*), and become less numerous with increasing silver content. Raman spectroscopy experiments on glasses along the Ag_*x*_(Ge_0.25_Se_0.75_)_(100−*x*)_ tie line also support an elimination of Se–Se bonds with increasing silver content: the intensity of the Se chain-mode at 250 cm^−1^ decreases with *x* increasing from zero, and is small or absent for *x*=25 [25]. The presence of Se–Se homopolar bonds has been suggested on the basis of previous anomalous X-ray scattering [21,34,35] and neutron diffraction [29] work, although the associated coordination numbers are larger than found in this study (table 2). They were not found, however, in a separate neutron diffraction experiment on the *x*=25 composition [24].

**Table 3.** The Ag–Se and Ag–Ag nearest-neighbour distances and coordination numbers extracted from neutron diffraction experiments on glasses along the Ag_*x*_(Ge_0.25_Se_0.75_)_(100−*x*)_ tie line. Results are also given from previous neutron diffraction [24,29] and anomalous X-ray scattering [21] experiments, from an analysis of anomalous X-ray scattering data using the reverse Monte Carlo method [34,35], and from a previous NDIS experiment on a related glass [36].

*x*	*r*_AgSe_ (Å)	*r*_AgSe_ (Å)	*r*_AgAg_ (Å)	${\bar{n}}_{\mathrm{Ag}}^{\mathrm{Se}}$	${\bar{n}}_{\mathrm{Ag}}^{\mathrm{Se}}$	${\bar{n}}_{\mathrm{Ag}}^{\mathrm{Ag}}$	function	reference
4.2	2.6	—	2.95	2.17	—	0.08	—	[35]
5	2.63(2)	2.75(2)	2.86(10)	2.1(2)	0.5(2)	1.7(3)	*D*_T,N_(*r*)	this work
	2.64(1)	2.83(2)	2.96(5)	2.1(2)	0.5(2)	1.7(3)	Δ*D*_Ag_(*r*)	this work
7.7^a^	2.65(1)	—	2.9(2)	3.5(1)	—	0.9(1)	Δ*G*_Ag_(*r*)	[36]
10	2.64(2)	2.75(2)	2.88(7)	2.2(2)	0.5(2)	1.6(3)	*D*_T,N_(*r*)	this work
15	2.65(2)	2.75(2)	2.91(5)	2.4(2)	0.7(1)	1.8(3)	*D*_T,N_(*r*)	this work
	2.74	—	3.07	2.6	—	4.7	—	[29]
20	2.62(2)	2.74(2)	2.94(5)	2.4(2)	0.7(1)	1.8(3)	*D*_T,N_(*r*)	this work
	2.6	—	2.95	2.80	—	0.45	—	[35]
25	2.63(2)	2.82(2)	2.95(5)	2.4(2)	0.8(2)	1.9(3)	*D*_T,N_(*r*)	this work
	2.63(1)	2.84(2)	3.03(5)	2.4(2)	0.8(2)	1.9(3)	Δ*D*_Ag_(*r*)	this work
	2.64(1)	3.14(2)	—	3.3(2)	0.6(2)	—	Δ*G*_Agμ_(*r*)	this work
	—	—	3.02(2)	—	—	1.9(2)	*g*_AgAg_(*r*)	this work
	2.68(1)	—	3.02(5)	3.0(1)	—	4.2(2)	—	[24]
	2.72	—	3.06	2.6	—	3.0	—	[29]
	2.62	—	3.35	3.9,4.6^b^	—	—	—	[21]
	2.65	—	3.05	2.66	—	0.45	—	[34]

^a^Corresponds to *x*=7.7 on the Ag_*x*_(Ge_0.23_Se_0.77_)_(100−*x*)_ tie line [36].^b^The values of 3.9 and 4.6 correspond to data analysis scenarios where the Se–Se coordination number is either 0.45 or zero, respectively.

### 4.2. Neutron diffraction with isotope substitution experiments

**Figure 8.**
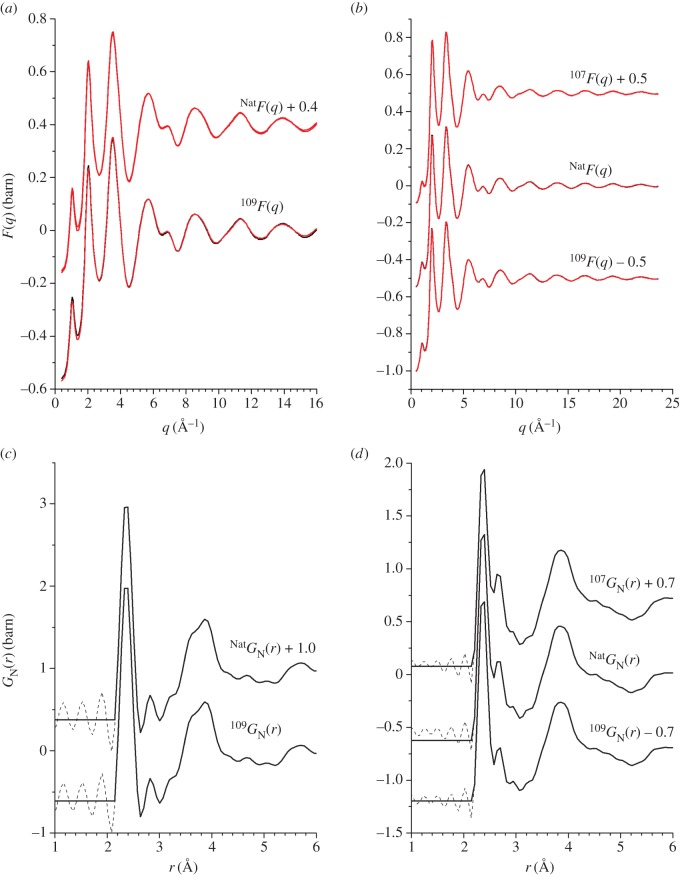
The measured total structure factors *F*(*q*) for the NDIS experiments on the (*a*) *x*=5 and (*b*) *x*=25 compositions. The solid (black) curves with vertical error bars give the measured functions, where the size of an error bar is smaller than the curve thickness at most *q*-values. The light solid (red) curves are the back Fourier transforms of the corresponding real-space functions *G*_N_(*r*) shown in (*c*) and (*d*), respectively, after the unphysical low-*r* oscillations shown by the broken curves are set to their theoretical $G_{N}(r\to 0)$ limit.

As emphasized by figure 7, there is overlap in *D*_T,N_(*r*) between the various *g*_*αβ*_(*r*) functions, which makes it valuable to apply the NDIS method. The measured total structure factors *F*(*q*) for the *x*=5 and *x*=25 compositions are shown in figure 8*a*,*b*, respectively, and the associated total pair-distribution functions *G*_N_(*r*) are shown in figure 8*c*,*d*, respectively. The latter reveal a growth in height of the Ag–Se peak at ≃2.64 Åwith magnitude of the silver scattering length (table 1).

**Figure 9.**
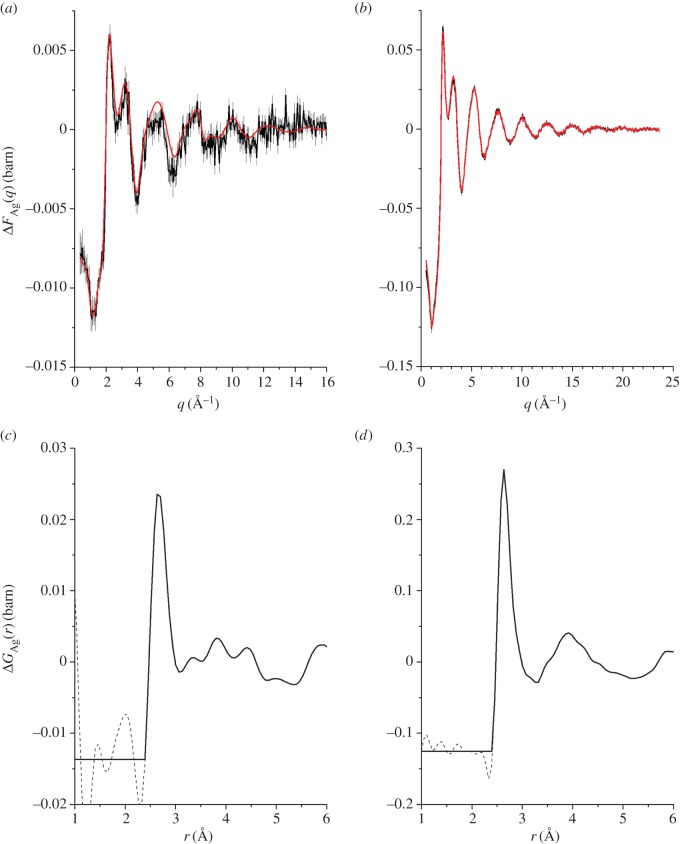
The measured difference functions (*a*) $\Delta F_{\mathrm{Ag}}(q){=}^{\mathrm{Nat}}F(k)-{}^{109}F(k)$ for *x*=5 and (*b*) $\Delta F_{\mathrm{Ag}}(q)={}^{107}F(k)-{}^{109}F(k)$ for *x*=25. The solid (black) curves with vertical error bars give the measured functions, and the light solid (red) curves are the back Fourier transforms of the corresponding Δ*G*_Ag_(*r*) functions shown in (*c*) and (*d*), respectively, after the unphysical low-*r* oscillations shown by the broken curves are set to their theoretical $\Delta G_{\mathrm{Ag}}(r\to 0)$ limit.

**Figure 10.**
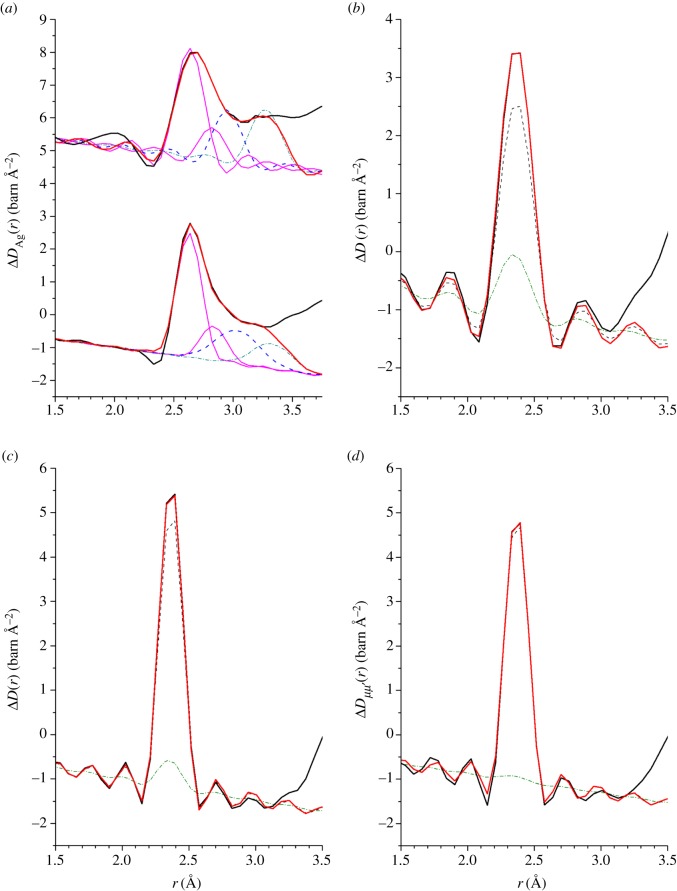
The fitted functions (*a*) Δ*D*_Ag_(*r*) for *x*=5 (top) and *x*=25 (bottom), (*b*) Δ*D*(*r*) for *x*=5, (*c*) Δ*D*(*r*) for *x*=25 and (*d*) Δ*D*_*μμ*^′^_(*r*) for *x*=25. The measured data sets are shown by the solid (black) curves, and the sums of fitted Gaussian functions are shown by the light solid (red) curves. In (*a*), the first two Gaussian functions represent Ag–Se correlations (solid (magenta) curves), the third Gaussian function represents Ag–Ag correlations (broken (blue) curve) and the fourth Gaussian function also represents Ag–Se correlations (chained (cyan) curve). In (*b*–*d*), the peak at 2.37 Å was fitted using two Gaussian functions, representing Ge–Se (broken (black) curve) and Se–Se (chained (green) curve) correlations, respectively.

The difference functions Δ*F*_Ag_(*q*), shown in figure 9*a*,*b* for the *x*=5 and *x*=25 compositions, respectively, reveal a measurable contrast between the total structure factors. The FSDP in *F*(*q*) becomes a trough at ≃1.08 Å^−1^ in Δ*F*_Ag_(*q*), and there is a slope in the difference function at small *q* that should develop into the small-angle scattering expected for phase-separated samples at smaller *q*-values. The corresponding real-space functions Δ*G*_Ag_(*r*) show an elimination of the μ–μ^′^ correlations at ≃2.37 Å, a first peak at ≃2.64 Å that originates from Ag–Se correlations, and indicate overlap between the Ag partial pair-distribution functions (figure 9*c*,*d*). To obtain additional information on the local structure, the first peak and shoulder in $\Delta D_{\mathrm{Ag}}(r)\equiv 4\pi n_{0}r\Delta G_{\mathrm{Ag}}(r)/|\Delta G_{\mathrm{Ag}}(r\to 0)|$ were fitted to a sum of Gaussian functions, each convoluted with *M*(*r*) [41]. The first and second Gaussian functions were attributed to Ag–Se correlations, the third Gaussian function was attributed to Ag–Ag correlations, and the fourth Gaussian function was also attributed to Ag–Se correlations. The fitted functions are shown in figure 10*a*, and the fitted parameters for the first three Gaussian functions are summarized in table 3. The fourth Gaussian function gave Ag–Se distances of 3.28(3) Å and 3.33(3) Å, and coordination numbers of ${\bar{n}}_{\mathrm{Ag}}^{\mathrm{Se}}=1.6(3)$ and ${\bar{n}}_{\mathrm{Ag}}^{\mathrm{Se}}=1.0(3)$ for the *x*=5 and *x*=25 compositions, respectively. In comparison, the shortest Ag–Ge distances are in the range 3.70–3.91 Åfor the crystalline polymorphs of Ag_8_GeSe_6_ [56–58]. Hence, the Ag–Se coordination number depends on the choice of cut-off distance. The first two fitted Gaussian functions give ${\bar{n}}_{\mathrm{Ag}}^{\mathrm{Se}}=2.6(3)$ for *x*=5 and ${\bar{n}}_{\mathrm{Ag}}^{\mathrm{Se}}=3.2(3)$ for *x*=25, values that increase to ${\bar{n}}_{\mathrm{Ag}}^{\mathrm{Se}}=4.2(4)$ for both compositions if the Ag–Se coordination number from the third fitted Gaussian function is included.

**Figure 11.**
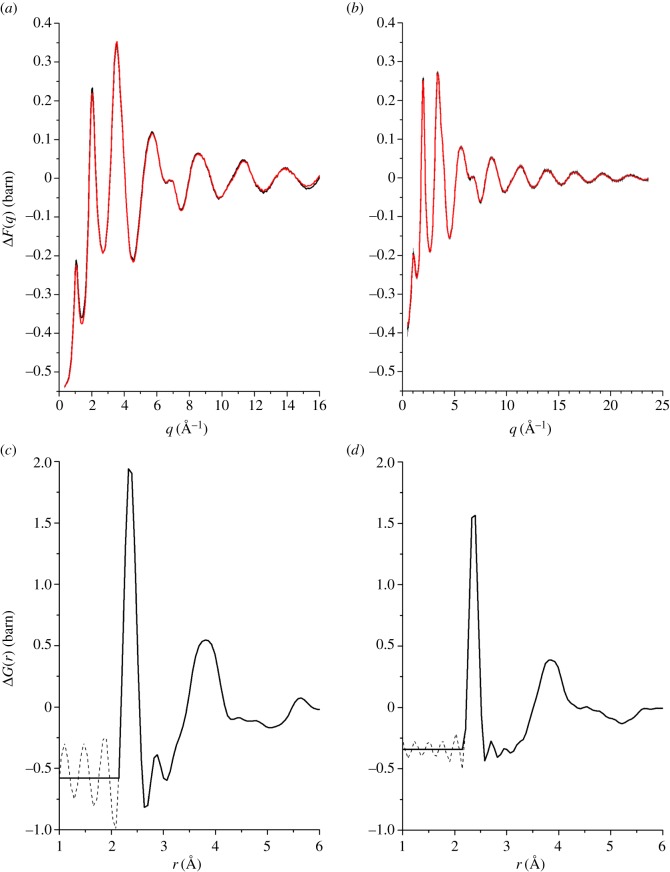
The measured difference functions (*a*) $\Delta F(q)=[b_{{\;}^{\mathrm{Nat}}\mathrm{Ag}}{}^{109}F(q)-b_{{}^{109}\mathrm{Ag}}{}^{\mathrm{Nat}}F(q)]/(b_{{\;}^{\mathrm{Nat}}\mathrm{Ag}}-b_{{}^{109}\mathrm{Ag}})$ for *x*=5 and (*b*) $\Delta F(q)=[b_{{}^{107}\mathrm{Ag}}{}^{109}F(q)-b_{{}^{109}\mathrm{Ag}}{}^{107}F(q)]/(b_{{}^{107}\mathrm{Ag}}-b_{{}^{109}\mathrm{Ag}})$ for *x*=25. The solid (black) curves with vertical error bars give the measured functions, and the light solid (red) curves are the back Fourier transforms of the corresponding Δ*G*(*r*) functions shown in (*c*) and (*d*), respectively, after the unphysical low-*r* oscillations shown by the broken curves are set to their theoretical ΔG(r→0) limit.

The difference functions Δ*F*(*q*) are shown in figure 11*a*,*b* for the *x*=5 and *x*=25 compositions, respectively, and the corresponding real-space functions Δ*G*(*r*) are shown in figure 11*c*,*d*, respectively. The Δ*G*(*r*) functions show an elimination of Ag–Se correlations at ≃2.64 Å. To obtain additional information on the local structure, the first peak in $\Delta D(r)\equiv 4\pi n_{0}r\Delta G(r)/|\Delta G(r\to 0)|$ was fitted to a sum of two Gaussian functions, each convoluted with *M*(*r*) [41], that were attributed to nearest-neighbour Ge–Se and Se–Se correlations, with the Ge–Se coordination number fixed at ${\bar{n}}_{\mathrm{Ge}}^{\mathrm{Se}}=4$. The results are shown in figure 10*b*,*c*, and the fitted μ–μ^′^ parameters are summarized in table 2. The latter are, within the experimental error, the same as those obtained from the fitted *D*_T,N_(*r*) functions.

**Figure 12.**
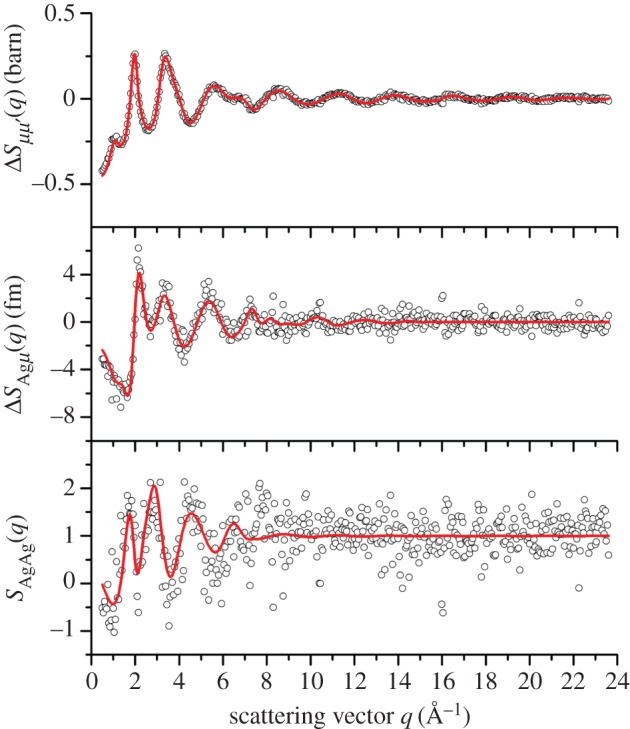
The partial structure factor *S*_AgAg_(*q*) and the difference functions Δ*S*_Agμ_(*q*) and Δ*S*_*μμ*^′^_(*q*) for the composition *x*=25. The circles give the measured data points, where the spread in values indicates the statistical uncertainty, and the solid (red) curves show the back Fourier transforms of the corresponding real-space data sets shown in figure 13 after the unphysical low-*r* oscillations are set to their theoretical low-*r* limit.

**Figure 13.**
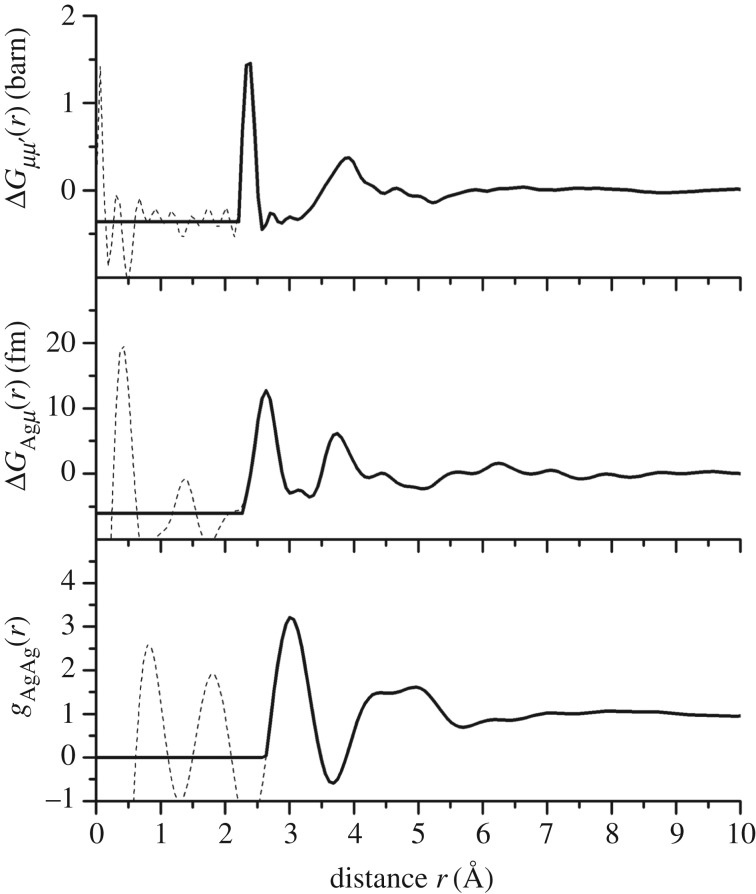
The partial pair-distribution function *g*_AgAg_(*r*) and the difference functions Δ*G*_Agμ_(*r*) and Δ*G*_*μμ*^′^_(*r*) for the composition *x*=25. The solid curves show the Fourier transforms of spline fits to the *q*-space data sets shown in figure 12, with the unphysical low-*r* oscillations (broken curves) set to their $g_{\mathrm{AgAg}}(r\to 0)$, $\Delta G_{\mathrm{Ag}\mu }(r\to 0)$ or $\Delta G_{\mu \mu^{\prime }}(r\to 0)$ limit.

For the *x*=25 composition, the partial structure factor *S*_AgAg_(*q*) and the difference functions Δ*S*_Agμ_(*q*) and Δ*S*_*μμ*^′^_(*q*) (figure 12) show that the FSDP in the total structure factors (figure 8*b*) originates from μ–μ^′^ correlations. The corresponding real-space functions are shown in figure 13, and the associated peak positions and coordination numbers are listed in tables 2 and 3. The effect of the modification function *M*(*r*) on *g*_AgAg_(*r*) and Δ*G*_Agμ_(*r*) is minimal, as indicated by an absence of pronounced oscillations in the convoluted Gaussian functions fitted to Δ*D*_Ag_(*r*) (figure 10*a*). The first peak in *g*_AgAg_(*r*) at 3.02(2) Åis well defined and gives a coordination number ${\bar{n}}_{\mathrm{Ag}}^{\mathrm{Ag}}=1.9(2)$. The first peak in Δ*G*_Agμ_(*r*) at 2.64(1) Å has a shoulder on its high-*r* side at 3.14(2) Å, and a coordination number ${\bar{n}}_{\mathrm{Ag}}^{\mathrm{Se}}=3.3(2)$ is obtained by integrating to the first minimum at 3.01 Å, or a value ${\bar{n}}_{\mathrm{Ag}}^{\mathrm{Se}}=3.9(3)$ is obtained by integrating to the second minimum at 3.31 Å. In comparison, the first peak in Δ*G*_*μμ*^′^_(*r*) at 2.37 Å is sharp, and its shape is affected by *M*(*r*). The first peak in $\Delta D_{\mu \mu^{\prime }}(r)\equiv 4\pi n_{0}r\Delta G_{\mu \mu^{\prime }}(r)/|\Delta G_{\mu \mu^{\prime }}(r\to 0)|$ was therefore fitted to a sum of two Gaussian functions, each convoluted with *M*(*r*) [41], that were attributed to Ge–Se and Se–Se bonds with ${\bar{n}}_{\mathrm{Ge}}^{\mathrm{Se}}=4$ (figure 10*d*). The fit yields a smaller Se–Se coordination number than obtained from *D*_T,N_(*r*) or Δ*D*(*r*) (table 2), which reflects a larger statistical error on Δ*S*_*μμ*^′^_(*q*).

## 5. Discussion

### 5.1. The Ag coordination environment

As indicated by figure 10*a*, there is overlap between the Ag–Se and Ag–Ag partial pair-distribution functions that contribute towards Δ*G*_Ag_(*r*). For the *x*=25 function, a broad distribution of nearest-neighbour Ag–Se distances is confirmed when Δ*G*_Ag_(*r*) is decomposed into its contributions from *g*_AgAg_(*r*) and Δ*G*_Agμ_(*r*) (figure 13). The latter gives a preferred Ag–Se bond distance of 2.64(1) Å, and a coordination number ${\bar{n}}_{\mathrm{Ag}}^{\mathrm{Se}}=3.3(2)$ for a cut-off distance of 3.01 Å, or ${\bar{n}}_{\mathrm{Ag}}^{\mathrm{Se}}=3.9(3)$ for a cut-off distance of 3.31 Å. For the *x*=5 composition, Δ*G*_Ag_(*r*) gives a preferred bond distance of 2.64(1) Å, and coordination numbers of ${\bar{n}}_{\mathrm{Ag}}^{\mathrm{Se}}=2.6(3)$ and ${\bar{n}}_{\mathrm{Ag}}^{\mathrm{Se}}=4.2(4)$ are obtained for similar cut-off distances. In comparison, for liquid Ag_2_Se where the full set of *g*_*αβ*_(*r*) functions is available from the NDIS method, there is also a broad distribution of Ag–Se distances and overlap between the Ag–Se and Ag–Ag partial pair-distribution functions [60]. The first peak in *g*_AgSe_(*r*) at 2.60(5) Åhas a shoulder on its high-*r* side and gives ${\bar{n}}_{\mathrm{Ag}}^{\mathrm{Se}}=9.3(5)$. The first peak in *g*_AgAg_(*r*) is at 2.80(5) Åand the corresponding coordination number ${\bar{n}}_{\mathrm{Ag}}^{\mathrm{Ag}}=5.3(5)$.

It should be noted that, in the analysis of the Δ*G*_Ag_(*r*) and Δ*G*_Agμ_(*r*) functions, the possibility of short Ag–Ge distances has been discounted. These neighbours have been found in first-principles molecular dynamics simulations of glasses along the Ag_*x*_(Ge_0.25_Se_0.75_)_(100−*x*)_ tie line, but are not particularly prevalent, i.e. there is a preference for Ag–Se bonds [28,32,33].

For the *x*=25 composition, Δ*G*_Ag_(*r*) and *g*_AgAg_(*r*) will provide information predominantly on the structure of the Ag-rich phase, and the latter gives a mean Ag–Ag distance ${\bar{r}}_{\mathrm{AgAg}}=3.02(2)$ Åwith ${\bar{n}}_{\mathrm{Ag}}^{\mathrm{Ag}}=1.9(2)$. For the *x*=5 composition, Δ*G*_Ag_(*r*) will also provide information predominantly on the structure of the Ag-rich phase, and it gives a mean distance ${\bar{r}}_{\mathrm{AgAg}}=2.96(5)$ Å with ${\bar{n}}_{\mathrm{Ag}}^{\mathrm{Ag}}=1.7(3)$. If these atoms reside predominantly in chain-like configurations in which the mean number of silver atoms is ${\bar{N}}_{c}$, then ${\bar{n}}_{\mathrm{Ag}}^{\mathrm{Ag}}=[2({\bar{N}}_{c}-2)+2]/{\bar{N}}_{c}$ or ${\bar{N}}_{c}=2/(2-{\bar{n}}_{\mathrm{Ag}}^{\mathrm{Ag}})$, giving chain lengths of ${\bar{N}}_{c}∼$20 and ${\bar{N}}_{c}∼$7 for the *x*=25 and *x*=5 compositions, respectively. Small Ag–Ag distances and low coordination numbers ${\bar{n}}_{\mathrm{Ag}}^{\mathrm{Ag}}<3$ have also been found for other silver-rich modified chalcogenide glasses by applying the NDIS method [61]. Examples include Ag_2_GeS_3_ where ${\bar{r}}_{\mathrm{AgAg}}≃$2.97 Å[62], AgAsS_2_ where ${\bar{r}}_{\mathrm{AgAg}}≃$3 Å [63], AgPS_3_ where ${\bar{r}}_{\mathrm{AgAg}}=2.9(1)$ Å and ${\bar{n}}_{\mathrm{Ag}}^{\mathrm{Ag}}=1.1(2)$ [48], Ag_2_As_3_Se_4_ where ${\bar{r}}_{\mathrm{AgAg}}=3.3(1)$ Å and ${\bar{n}}_{\mathrm{Ag}}^{\mathrm{Ag}}=2.7(2)$ [64], and AgAsTe_2_ where ${\bar{r}}_{\mathrm{AgAg}}=3.03(2)$ Å and ${\bar{n}}_{\mathrm{Ag}}^{\mathrm{Ag}}=2.8(4)$ [65]. Similar findings apply to the copper-rich modified chalcogenide glass Cu_2_As_3_Se_4_ where ${\bar{r}}_{\mathrm{CuCu}}=2.70(4)$ Å and ${\bar{n}}_{\mathrm{Cu}}^{\mathrm{Cu}}=1.0(3)$ [64,66].

Overall, the results show a structure for the Ag-rich phase that changes with composition along the Ag_*x*_(Ge_0.25_Se_0.75_)_(100−*x*)_ tie line. This observation is consistent with EFM results that show changes to the electric permittivity of the Ag-rich (and Ag-poor) phase with change of *x* [12]. Conductive atomic force microscopy (C-AFM) experiments show an increase with *x* in the electrical conductivity of the Ag-rich phase for *x*≥10, i.e. the results are consistent with a structure for the Ag-rich phase that continues to evolve as silver is added to the base glass [11]. The C-AFM results show a small or negligible electrical conductivity for the Ag-poor phase.

### 5.2. Model for the structure of the modified glass

What happens to the structure of the Se-rich base glass as silver is added? Here, a starting point is provided by the ‘8-N’ rule for the base glass, where the overall coordination numbers of Ge and Se are *Z*_Ge_=4 and *Z*_Se_=2, respectively. A chemically ordered network model appears to hold for glasses such as Ge_0.25_Se_0.75_ and Ge_0.20_Se_0.80_, as supported by the full set of *g*_*αβ*_(*r*) functions measured for these materials by using the NDIS method [55]. Hence, if the numbers of Ge and Se atoms in the base glass are denoted by *N*_Ge_ and *N*_Se_, respectively, the number of Se–Se bonds can be enumerated as $N_{\mathrm{SeSe}}=N_{\mathrm{Se}}Z_{\mathrm{Se}}/2-N_{\mathrm{Ge}}Z_{\mathrm{Ge}}/2$ [36]. The corresponding coordination number for Se–Se homopolar bonds is given by ${\bar{n}}_{\mathrm{Se}}^{\mathrm{Se}}=2N_{\mathrm{SeSe}}/N_{\mathrm{Se}}=Z_{\mathrm{Se}}-Z_{\mathrm{Ge}}c_{\mathrm{Ge}}/c_{\mathrm{Se}}$, equivalent to the expectation of a chemically ordered network model for a Se-rich Ge–Se base glass [67].

**Figure 14.**
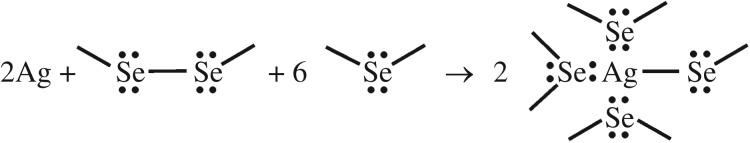
Schematic of a bonding scheme in which two Ag atoms break a single Se–Se homopolar bond to create two fourfold coordinated Ag atoms. Following the proposal of Kastner [68], each AgSe_4_ motif contains one covalent Ag–Se bond, formed by using the valence electron from Ag and a valence electron from one of the Se atoms of the initial homopolar bond, and three dative Ag–Se covalent bonds, formed by using the lone pair electrons (shown by dots) on three other Se atoms and the three empty *s*-*p* orbitals of the Ag atom.

When a monovalent metal such as silver is added to the Se-rich Ge_0.25_Se_0.75_ base glass, the metal atoms are expected to bond preferentially to Se, so that Se–Se homopolar bonds are removed. For example, in the bonding scheme of Kastner [68], where the covalent contribution to the bonding is significant and the effect of electronic *d* states can be neglected, each silver atom is fourfold coordinated by Se atoms. One of the Ag–Se bonds is formed by using the valence electron from Ag and a valence electron from Se, and the other three Ag–Se bonds are dative, using lone-pair electrons on three other Se atoms (figure 14). In consequence, one Se atom remains twofold coordinated, in accordance with the ‘8-N’ rule, whereas the other three Se atoms become threefold coordinated. A single added Ag atom will break a single Se–Se bond to combine with one of these Se atoms and leave the other Se atom with a dangling bond, whereas two added Ag atoms will break a single Se–Se bond without leaving a dangling bond. The mean number of broken Se–Se bonds per silver atom is therefore ${\bar{N}}_{\mathrm{broken}}=1$ or ${\bar{N}}_{\mathrm{broken}}=0.5$, respectively. The reduced number of Se–Se bonds is given by $N_{\mathrm{SeSe}}=N_{\mathrm{Se}}Z_{\mathrm{Se}}/2-N_{\mathrm{Ge}}Z_{\mathrm{Ge}}/2-N_{\mathrm{Ag}}{\bar{N}}_{\mathrm{broken}}$, and the associated coordination number for this modified chemically ordered network (MCON) model is given by

5.1 $${\bar{n}}_{\mathrm{Se}}^{\mathrm{Se}}=2N_{\mathrm{SeSe}}/N_{\mathrm{Se}}=Z_{\mathrm{Se}}-Z_{\mathrm{Ge}}c_{\mathrm{Ge}}/c_{\mathrm{Se}}-2c_{\mathrm{Ag}}{\bar{N}}_{\mathrm{broken}}/c_{\mathrm{Se}}.$$

**Figure 15.**
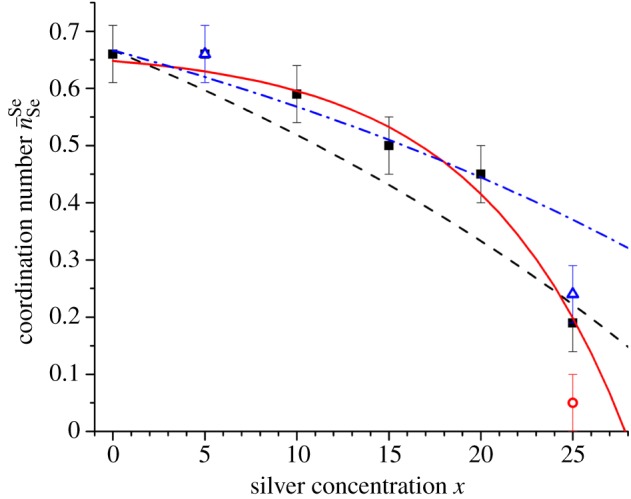
The composition dependence of ${\bar{n}}_{\mathrm{Se}}^{\mathrm{Se}}$ as obtained by fitting the first peak in *D*_T,N_(*r*) (filled squares), the first peak in Δ*D*(*r*) for *x*=5 or *x*=25 (open (blue) triangles), or the first peak in Δ*D*_*μμ*^′^_(*r*) for *x*=25 (open (red) circle). The silver concentration *x* is identical to *c*_Ag_, provided the latter is expressed as a percentage. The solid (red) curve was obtained from a least-squares fit to the filled squares, and corresponds to ${\bar{n}}_{\mathrm{Se}}^{\mathrm{Se}}=0.67-0.022\exp(x/8.11)$. The fit gives ${\bar{n}}_{\mathrm{Se}}^{\mathrm{Se}}=0$ at *x*≃28. The expectations of the MCON for ${\bar{N}}_{\mathrm{broken}}=0.5$ (broken (black) curve) and ${\bar{N}}_{\mathrm{broken}}=1/3$ (chained (blue) curve) are also given.

In figure 15, the measured values of ${\bar{n}}_{\mathrm{Se}}^{\mathrm{Se}}$ for glasses along the Ag_*x*_(Ge_0.25_Se_0.75_)_(100−*x*)_ tie line are compared to the expectations of the MCON model for two different values of ${\bar{N}}_{\mathrm{broken}}$. The MCON with a single value of ${\bar{N}}_{\mathrm{broken}}$ does not account for the full composition dependence of ${\bar{n}}_{\mathrm{Se}}^{\mathrm{Se}}$. Instead, the model suggests that fewer Se–Se bonds are broken (${\bar{N}}_{\mathrm{broken}}=1/3$) than expected from the bonding scheme proposed by Kastner [68] (${\bar{N}}_{\mathrm{broken}}=0.5$) at most compositions, i.e. there is a greater propensity for Ag to form dative bonds with Se. At *x* = 25, the measured value of ${\bar{n}}_{\mathrm{Se}}^{\mathrm{Se}}$ does, however, coincide with the prediction of the MCON for ${\bar{N}}_{\mathrm{broken}}=0.5$, i.e. the structure of this silver-rich phase is consistent with a bonding scheme in which the addition of two Ag atoms eliminates a single Se–Se bond (figure 14). The experimental results shown in figure 15 indicate an elimination of all the Se–Se homopolar bonds by the addition of silver when *x*≃28. This concentration corresponds to the limit of glass forming ability along the Ag_*x*_(Ge_0.25_Se_0.75_)_(100−*x*)_ tie line (figure 1), i.e. glass formation is related to the availability of Se–Se homopolar bonds.

It should be noted that electronic *d* states may play an important role in the bonding in Ag(I) glasses. For example, an investigation of the relative stability of threefold versus fourfold coordination complexes using a molecular orbital approach suggests that the lower-coordination-number conformation can be stabilized over the regular tetrahedral arrangement if there is a distortion via a second-order Jahn–Teller effect wherein the *d* orbitals of the occupied outer shell are mixed with the *s* orbital of the valence shell [69].

## 6. Conclusion

The structural changes associated with the transition from a semiconductor to a fast-ion conductor with increasing silver content along the Ag_*x*_(Ge_0.25_Se_0.75_)_(100−*x*)_ tie line were investigated by combining the methods of EFM, X-ray diffraction, and neutron diffraction. The microscopy results show phase separation into silver-rich and silver-poor phases, and are consistent with a percolation of the Ag-rich phase at the onset of fast-ion conductivity when *x*≃8. The NDIS results show that the evolution with composition in the structure of the Ag-rich phase indicated by EFM and C-AFM experiments [11,12] is associated with a change to the coordination environment of silver in which the number of Ag–Se nearest-neighbours, distributed about a distance of 2.64(1) Å, increases from 2.6(3) at *x*=5 to 3.3(2) at *x*=25. The Ag–Ag nearest-neighbour coordination number is 1.7(3) for a distance of 2.96(5) Åat *x*=5 versus 1.9(2) for a distance of 3.02(2) Åat *x*=25. The diffraction results are consistent with the presence of GeSe_4_ tetrahedra for all of the glass compositions, and indicate a breakage of Se–Se homopolar bonds as silver is added to the Se-rich base glass. The limit of glass formation along the tie line at *x*≃28 coincides with an elimination of these homopolar bonds.

## Data accessibility

The data sets created during this research are openly available from the University of Bath data archive at https://doi.org/10.15125/BATH-00423 [70].

## References

[1] Borisova ZU 1981 *Glassy semiconductors*. New York, NY: Plenum Press.

[2] Kawasaki M, Kawamura J, Nakamura Y, Aniya M 1999 Ionic conductivity of Ag_*x*_(GeSe_3_)_1−*x*_ (0≤*x*≤0.571) glasses. *Solid State Ion.* 123, 259–269. (doi:10.1016/S0167-2738(99)00117-4)

[3] Mirandou M, Fontana M, Arcondo B 2003 DC conductivity of GeSeAg glasses at room temperature. *J. Mater. Process. Tech.* 143–144, 420–424. (doi:10.1016/S0924-0136(03)00437-0)

[4] Ureña MA, Piarristeguy AA, Fontana M, Arcondo B 2005 Ionic conductivity (Ag^+^) in AgGeSe glasses. *Solid State Ion.* 176, 505–512. (doi:10.1016/j.ssi.2004.09.008)

[5] Arcondo B, Ureña MA, Piarristeguy A, Pradel A, Fontana M 2007 Homogeneous-inhomogeneous models of Ag_*x*_(Ge_0.25_Se_0.75_)_100−*x*_ bulk glasses. *Physica B* 389, 77–82. (doi:10.1016/j.physb.2006.07.028)

[6] Piarristeguy A, Conde Garrido JM, Ureña MA, Fontana M, Arcondo B 2007 Conductivity percolation transition of Ag_*x*_(Ge_0.25_Se_0.75_)_100−*x*_ glasses. *J. Non-Cryst. Solids* 353, 3314–3317. (doi:10.1016/j.jnoncrysol.2007.05.078)

[7] Ureña MA, Fontana M, Piarristeguy A, Arcondo B 2010 AgGeSe-based bulk glasses: a survey of their fundamental properties. *J. Alloys Compd.* 495, 305–308. (doi:10.1016/j.jallcom.2009.11.081)

[8] Balan V, Piarristeguy A, Ramonda M, Pradel A, Ribes M 2006 Phase separation and ionic conductivity: an electric force microscopy investigation of silver chalcogenide glasses. *J. Optoelectron. Adv. Mater.* 8, 2112–2116.

[9] Arcondo B, Ureña MA, Piarristeguy A, Pradel A, Fontana M 2007 Nanoscale intrinsic heterogeneities in Ag–Ge–Se glasses and their correlation with physical properties. *Appl. Surface Sci.* 254, 321–324. (doi:10.1016/j.apsusc.2007.07.094)

[10] Piarristeguy A, Ramonda M, Ureña A, Pradel A, Ribes M 2007 Phase separation in Ag–Ge–Se glasses. *J. Non-Cryst. Solids* 353, 1261–1263. (doi:10.1016/j.jnoncrysol.2006.09.065)

[11] Piarristeguy AA, Ramonda M, Frolet N, Ribes M, Pradel A 2010 High resolution electrical characterisation of Ag-conducting heterogeneous chalcogenide glasses. *Solid State Ion.* 181, 1205–1208. (doi:10.1016/j.ssi.2010.06.050)

[12] Piarristeguy AA, Ramonda M, Pradel A 2010 Local electrical characterization of Ag conducting chalcogenide glasses using electric force microscopy. *J. Non-Cryst. Solids* 356, 2402–2405. (doi:10.1016/j.jnoncrysol.2010.03.024)

[13] Mitkova M, Kozicki MN 2002 Silver incorporation in Ge–Se glasses used in programmable metallization cell devices. *J. Non-Cryst. Solids* 299–302, 1023–1027. (doi:10.1016/S0022-3093(01)01068-7)

[14] Kozicki MN, Mitkova M, Park M, Balakrishnan M, Gopalan C 2003 Information storage using nanoscale electrodeposition of metal in solid electrolytes. *Superlattices Microstruct.* 34, 459–465. (doi:10.1016/j.spmi.2004.03.042)

[15] Kozicki MN, Gopalan V, Balakrishnan M, Park M, Mitkova M 2004 Non-volatile memory based on solid electrolytes. In *Proc. 2004 IEEE Computational Systems Bioinformatics Conf., Stanford, CA, USA*, pp. 10–17 (doi:10.1109/NVMT.2004.1380792).

[16] Kozicki MN, Park M, Mitkova M 2005 Nanoscale memory elements based on solid-state electrolytes. *IEEE Trans. Nanotechnol.* 4, 331–338. (doi:10.1109/TNANO.2005.846936)

[17] Drabold DA 2009 Topics in the theory of amorphous materials. *Eur. Phys. J. B* 68, 1–21. (doi:10.1140/epjb/e2009-00080-0)

[18] Dandamudi P, Kozicki MN, Barnaby HJ, Gonzalez-Velo Y, Mitkova M, Holbert KE, Ailavajhala M, Yu W 2013 Sensors based on radiation-induced diffusion of silver in germanium selenide glasses. *IEEE Trans. Nucl. Sci.* 60, 4257–4264. (doi:10.1109/TNS.2013.2285343)

[19] Prasai K, Drabold DA 2014 Simulations of silver-doped germanium-selenide glasses and their response to radiation. *Nanoscale Res. Lett.* 9, 594 (doi:10.1186/1556-276X-9-594) 25426005 10.1186/1556-276X-9-594 PMC4240743

[20] Fischer-Colbrie A, Bienenstock A, Fuoss PH, Marcus MA 1988 Structure and bonding in photodiffused amorphous Ag–GeSe_2_ thin films. *Phys. Rev. B* 38, 12 388–12 403. (doi:10.1103/PhysRevB.38.12388) 10.1103/physrevb.38.12388 9946180

[21] Westwood JD, Georgopoulos P, Whitmore DH 1988 Structure of glassy fast ion conductors: differential anomalous x-ray scattering study of a Ag–Ge–Se glass using synchrotron radiation. *J. Non-Cryst. Solids* 107, 88–100. (doi:10.1016/0022-3093(88)90097-X)

[22] Westwood JD, Georgopoulos P 1989 A maximum entropy method of determining the partial distribution functions of multicomponent amorphous materials. *J. Non-Cryst. Solids* 108, 169–179. (doi:10.1016/0022-3093(89)90580-2)

[23] Dejus RJ, Susman S, Volin KJ, Price DL, Montague DG 1988 The structure of silver/germanium/selenide glass. *J. Non-Cryst. Solids* 106, 34–37. (doi:10.1016/0022-3093(88)90222-0)

[24] Dejus RJ, Susman S, Volin KJ, Montague DG, Price DL 1992 Structure of vitreous Ag–Ge–Se. *J. Non-Cryst. Solids* 143, 162–180. (doi:10.1016/S0022-3093(05)80565-4)

[25] Mitkova M, Wang Y, Boolchand P 1999 Dual chemical role of Ag as an additive in chalcogenide glasses. *Phys. Rev. Lett.* 83, 3848–3851. (doi:10.1103/PhysRevLett.83.3848)

[26] Iyetomi H, Vashishta P, Kalia RK 2000 Incipient phase separation in Ag/Ge/Se glasses: clustering of Ag atoms. *J. Non-Cryst. Solids* 262, 135–142. (doi:10.1016/S0022-3093(99)00692-4)

[27] Červinka L, Bergerová J, Tichý L, Rocca F 2005 A contribution to the structure of Ge–Se–Ag glasses. *Phys. Chem. Glasses* 46, 444–450.

[28] Tafen DN, Drabold DA, Mitkova M 2005 Silver transport in Ge_*x*_Se_1−*x*_:Ag materials: ab initio simulation of a solid electrolyte. *Phys. Rev. B* 72, 054206 (doi:10.1103/PhysRevB.72.054206)

[29] Cuello GJ, Piarristeguy AA, Fernández-Martínez A, Fontana M, Pradel A 2007 Structure of chalcogenide glasses by neutron diffraction. *J. Non-Cryst. Solids* 353, 729–732. (doi:10.1016/j.jnoncrysol.2006.12.036)

[30] Piarristeguy A, Mirandou M, Fontana M, Arcondo B 2000 X-ray analysis of GeSeAg glasses. *J. Non-Cryst. Solids* 273, 30–35. (doi:10.1016/S0022-3093(00)00141-1)

[31] Kumara LSR, Ohara K, Kawakita Y, Jóvári P, Hidaka M, Sung NE, Beuneu B, Takeda S 2011 Local structure of superionic glass Ag_*x*_(GeSe_3_)_1−*x*_, *x*=0.565. *EPJ Web Conf.* 15, 02007 (doi:10.1051/epjconf/20111502007)

[32] Prasai B, Drabold DA 2011 *Ab initio* simulation of solid electrolyte materials in liquid and glassy phases. *Phys. Rev. B* 83, 094202 (doi:10.1103/PhysRevB.83.094202)

[33] Piarristeguy AA, Cuello GJ, Fernández-Martínez A, Cristiglio V, Johnson M, Ribes M, Pradel A 2012 Short range order and Ag diffusion threshold in Ag_*x*_(Ge_0.25_Se_0.75_)_100−*x*_ glasses. *Phys. Status Solidi B* 249, 2028–2033. (doi:10.1002/pssb.201200384)

[34] Stellhorn JR, Hosokawa S, Kawakita Y, Gies D, Pilgrim W-C, Hayashi K, Ohoyama K, Blanc N, Boudet N 2016 Local structure of room-temperature superionic Ag–GeSe_3_ glasses. *J. Non-Cryst. Solids* 431, 68–71. (doi:10.1016/j.jnoncrysol.2015.02.027)

[35] Stellhorn JR, Hosokawa S, Pilgrim W-C, Kawakita Y, Kamimura K, Kimura K, Blanc N, Boudet N 2016 Structural aspects of the superionic conduction mechanism in Ag–GeSe_3_ glasses. *Z. Phys. Chem.* 230, 369–386. (doi:10.1515/zpch-2015-0667)

[36] Zeidler A, Salmon PS, Piarristeguy A, Pradel A, Fischer HE 2016 Structure of glassy Ag–Ge–Se by neutron diffraction with isotope substitution. *Z. Phys. Chem.* 230, 417–432. (doi:10.1515/zpch-2015-0727)

[37] Pandey A, Biswas P, Drabold DA 2016 Inversion of diffraction data for amorphous materials. *Sci. Rep.* 6, 33731 (doi:10.1038/srep33731) 27652893 10.1038/srep33731 PMC5031976

[38] Borisova ZU, Rykova TS, Turkina EY, Tabolin AR 1984 Interaction and glass formation in the system Ge–Se–Ag. *Inorg. Mater. (USSR)* 20, 1796–1800.

[39] Fischer HE, Barnes AC, Salmon PS 2006 Neutron and x-ray diffraction studies of liquids and glasses. *Rep. Prog. Phys.* 69, 233–299. (doi:10.1088/0034-4885/69/1/R05)

[40] Faber TE, Ziman JM 1965 A theory of the electrical properties of liquid metals III. The resistivity of binary alloys. *Phil. Mag.* 11, 153–173. (doi:10.1080/14786436508211931)

[41] Martin RA, Salmon PS, Fischer HE, Cuello GJ 2003 Structure of dysprosium and holmium phosphate glasses by the method of isomorphic substitution in neutron diffraction. *J. Phys.: Condens. Matter* 15, 8235–8252. (doi:10.1088/0953-8984/15/49/003)

[42] Sears VF 1992 Neutron scattering lengths and cross sections. *Neutron News* 3, 26–37. (doi:10.1080/10448639208218770)

[43] Piarristeguy A, Fontana M, Arcondo B 2003 Structural considerations about the (Ge_0.25_Se_0.75_)_100−*x*_Ag_*x*_ glasses. *J. Non-Cryst. Solids* 332, 1–10. (doi:10.1016/j.jnoncrysol.2003.09.011)

[44] Ureña MA, Fontana M, Arcondo B, Clavaguera-Mora MT 2003 Crystallization processes of Ag–Ge–Se superionic glasses. *J. Non-Cryst. Solids* 320, 151–167. (doi:10.1016/S0022-3093(03)00022-X)

[45] Wang Y, Mitkova M, Georgiev DG, Mamedov S, Boolchand P 2003 Macroscopic phase separation of Se-rich (*x*<1/3) ternary Ag_*y*_(Ge_*x*_Se_1−*x*_)_1−*y*_ glasses. *J. Phys.: Condens. Matter* 15, S1573–S1584. (doi:10.1016/S0022-3093(03)00022-X)

[46] Fischer HE, Cuello GJ, Palleau P, Feltin D, Barnes AC, Badyal YS, Simonson JM 2002 D4c: a very high precision diffractometer for disordered materials. *Appl. Phys. A* 74, S160–S162. (doi:10.1007/s003390101087)

[47] Salmon PS, Zeidler A, Fischer HE 2016 Optimizing the counting times for sample-in-container scattering experiments. *J. Appl. Cryst.* 49, 2249–2251. (doi:10.1107/S160057671601493X)

[48] Salmon PS, Xin S, Fischer HE 1998 Structure of the glassy fast-ion conductor AgPS_3_ by neutron diffraction. *Phys. Rev. B* 58, 6115–6123. (doi:10.1103/PhysRevB.58.6115)

[49] Hammersley AP, Svensson SO, Hanfland M, Fitch AN, Häusermann D 1996 Two-dimensional detector software: from real detector to idealised image or two-theta scan. *High Press. Res.* 14, 235–248. (doi:10.1080/08957959608201408)

[50] Hammersley AP 1997 FIT2D: an introduction and overview. ESRF Internal Report. ESRF97HA02T.

[51] Waasmaier D, Kirfel A 1995 New analytical scattering-factor functions for free atoms and ions. *Acta Cryst. A* 51, 416–431. (doi:10.1107/S0108767394013292)

[52] Cromer DT, Mann JB 1967 Compton scattering factors for spherically symmetric free atoms. *J. Chem. Phys.* 47, 1892–1893. (doi:10.1063/1.1712213)

[53] Cromer DT 1969 Compton scattering factors for aspherical free atoms. *J. Chem. Phys.* 50, 4857–4859. (doi:10.1063/1.1670980)

[54] Zeidler A, Salmon PS 2016 Pressure-driven transformation of the ordering in amorphous network-forming materials. *Phys. Rev. B* 93, 214204 (doi:10.1103/PhysRevB.93.214204)

[55] Rowlands RF, Zeidler A, Fischer HE, Salmon PS Submitted. Structure of amorphous GeSe_3_ and GeSe_4_ by neutron diffraction with isotope substitution.

[56] Gorochov O 1968 The compounds Ag_8_MX_6_ (M = Si, Ge, Sn; X = S, Se, Te). *Bull. Soc. Chim. France* 1968, 2263–2275.

[57] von Unterrichter J, Range K-J 1978 Ag_8_GeTe_6_, a representative of the argyrodite family. *Z. Naturforsch. B* 33, 866–872. (doi:10.1107/S0567740880003032)

[58] Carré D, Ollitrault-Fichet R, Flahaut J 2008 Structure de Ag_8_GeSe_6_*β*^′^. *Acta Cryst. B* 36, 245–249. (doi:10.1107/S0567740880003032)

[59] Dejus RJ, LePoire DJ, Susman S, Volin KJ, Price DL 1991 Dynamics of vitreous Ag–Ge–Se. *Phys. Rev. B* 44, 11 705–11 713. (doi:10.1103/PhysRevB.44.11705) 10.1103/physrevb.44.11705 9999304

[60] Barnes AC, Lague SB, Salmon PS, Fischer HE 1997 A determination of the structure of liquid Ag_2_Se using neutron diffraction and isotopic substitution. *J. Phys.: Condens. Matter* 9, 6159–6173. (doi:10.1088/0953-8984/9/29/002)

[61] Salmon PS, Liu J 1996 The coordination environment of Ag and Cu in ternary chalcogenide glasses. *J. Non-Cryst. Solids* 205–207, 172–175. (doi:10.1016/S0022-3093(96)00225-6)

[62] Lee JH, Owens AP, Pradel A, Hannon AC, Ribes M, Elliott SR 1996 Structure determination of Ag–Ge–S glasses using neutron diffraction. *Phys. Rev. B* 54, 3895–3909. (doi:10.1103/PhysRevB.54.3895) 10.1103/physrevb.54.3895 9986289

[63] Penfold IT, Salmon PS 1990 Glass formation and short-range order in chalcogenide materials: the (Ag_2_S)_*x*_(As_2_S_3_)_1−*x*_ (0≤*x*≤1) pseudobinary tie line. *Phys. Rev. Lett.* 64, 2164–2167. (doi:10.1103/PhysRevLett.64.2164) 10041600 10.1103/PhysRevLett.64.2164

[64] Benmore CJ, Salmon PS 1994 Structure of fast ion conducting and semiconducting chalcogenide alloys. *Phys. Rev. Lett.* 73, 264–267. (doi:10.1103/PhysRevLett.73.264) 10057126 10.1103/PhysRevLett.73.264

[65] Liu J, Salmon PS 1997 Structural ordering in Ag-based ternary chalcogenide glasses. *Europhys. Lett.* 39, 521–526. (doi:10.1103/PhysRevB.58.6115)

[66] Xin S, Liu J, Salmon PS 2008 Structure of Cu–As–Se glasses investigated by neutron diffraction with copper isotope substitution. *Phys. Rev. B* 78, 064207 (doi:10.1103/PhysRevB.78.064207)

[67] Salmon PS 2007 Structure of liquids and glasses in the Ge–Se binary system. *J. Non-Cryst. Solids* 353, 2959–2974. (doi:10.1016/j.jnoncrysol.2007.05.152)

[68] Kastner M 1978 Prediction of the influence of additives on the density of valence-alternation centres in lone-pair semiconductors. *Phil. Mag. B* 37, 127–133. (doi:10.1080/13642817808245313)

[69] Burdett JK, Eisenstein O 1992 From three- to four-coordination in copper(I) and silver(I). *Inorg. Chem.* 31, 1758–1762. (doi:10.1021/ic00036a007)

[70] Zeidler A, Salmon PS, Whittaker DAJ, Piarristeguy A, Pradel A, Fischer HE, Benmore CJ, Gulbiten O 2018 Data from: Structure of semiconducting versus fast-ion conducting glasses in the Ag–Ge–Se system University of Bath (https://doi.org/10.15125/BATH-00423) 10.1098/rsos.171401 PMC5792920 29410843

